# Heart Rate Variability Biofeedback Training Can Improve Menopausal Symptoms and Psychological Well-Being in Women with a Diagnosis of Primary Breast Cancer: A Longitudinal Randomized Controlled Trial

**DOI:** 10.3390/curroncol32030150

**Published:** 2025-03-04

**Authors:** Karina Dolgilevica, Elizabeth Grunfeld, Nazanin Derakshan

**Affiliations:** 1 Department of Psychological Sciences, Birkbeck, University of London, London WC1E 7HX, UK; kdolgi01@student.bbk.ac.uk (K.D.); e.grunfeld@bbk.ac.uk (E.G.); 2 Resilience and Post-Traumatic Growth Centre, National Centre for Integrative Oncology (NCIO), Reading RG10 9XQ, UK

**Keywords:** breast cancer, heart rate variability, menopausal symptoms, intervention

## Abstract

Breast cancer survivors experience numerous chronic symptoms linked to autonomic dysfunction including anxiety, stress, insomnia, menopausal symptoms, and cognitive impairment. Effective non-pharmacological solutions to address these are currently lacking. Methods: Our three-armed longitudinal randomized controlled trial assessed the effectiveness of a 4-week remote smartphone-based heart rate variability biofeedback intervention which involved daily paced breathing at 6 breaths p/min; active (12 breaths p/min) and waitlist controls were included. Heart rate variability and self-reported cancer-related symptoms were assessed at baseline, post-, and 6 months-post intervention. Participants were 60 UK-based women with primary breast cancer history (6 to 60 months post-active treatment). Results: The intervention group showed significant increases in low-frequency heart rate variability over time (F (4, 103.89) = 2.862, *p* = 0.027, *d* = 0.33), long-lasting improvement in sleep quality (F (4, 88.04) = 4.87, *p* = 0.001, *d* = 0.43) and cessations in night sweats (*X*^2^ (2, *N* = 59) = 6.44, *p* = 0.04, Cramer’s V = 0.33), and reduced anxiety post-intervention compared to the active and waitlist controls (F (4, 82.51) = 2.99, *p* = 0.023, *d* = 0.44). Other findings indicated that the intervention and active control participants reported lasting improvements in cognitive function, fatigue, and stress-related symptoms (all *ps* < 0.05). The waitlist group reported no symptom changes across time. Conclusion: Heart rate variability biofeedback is a feasible intervention for addressing diverse chronic symptoms commonly reported by breast cancer survivors.

## 1. Introduction

Breast cancer is the most diagnosed cancer in the world with almost 2.3 million new female breast cancer cases recorded in 2020 [1]. It is associated with a host of life-altering psychological and physical side-effects, which can continue affecting women for many years post-diagnosis.

Breast cancer survivors typically experience multiple physical and mental health issues, with combinations of symptoms co-occurring at the same time. Some of the most common health issues experienced throughout survivorship include post-traumatic stress [2], depression and anxiety [3], fear of cancer recurrence [4], cancer-related cognitive impairment [5], fatigue and pain [6], osteoporosis [7], menopausal symptoms, such as hot flushes and night sweats, and insomnia [8]. Breast cancer survivors are also at an increased risk of cardiovascular health problems [9].

The wide-ranging symptoms observed in breast cancer survivors are linked to an overactive sympathetic nervous system, where the overactivation is likely to be promoted by multidimensional mechanisms [10]. It is well-established that chronic stress exposure can lead to the sensitization of the body’s inflammatory stress response, which is linked to cardiovascular, immune, neurological, and other pathologies [11]. Estrogen depletion associated with menopause and psychological distress is also linked to challenges in restoring the autonomic nervous system balance in cancer survivors [12]. This autonomic dysregulation is reflected in the cardiovascular function and can be accessed via the heart rate, specifically, via heart rate variability.

Heart rate variability is a measure of variation in time intervals between consecutive heart beats which can be measured in milliseconds during a continuous heart rate recording [13]. Heart rate variability reflects the capacity of the nervous system to maintain its functions in balance [14], where higher baseline heart rate variability is associated with better cardiovascular, cognitive, and emotional functioning [15,16]. Lower heart rate variability, on the other hand, is associated with higher levels of stress and anxiety [17,18], poor health and higher mortality across populations [19]. Women with a breast cancer diagnosis typically show reduced baseline heart rate variability, which is associated with poor health outcomes [20,21] and greater mortality risks [22]. Interventions which can increase heart rate variability might, therefore, be beneficial for enhancing autonomic balance and improving well-being in breast cancer.

Heart rate variability biofeedback is a promising intervention which involves performing slow paced breathing at ~6 breaths per minute (~0.1 Hz), that is, resonance frequency with feedback of real-time heart rate variability changes [13]. This technique allows people to alter their heart rate activity which is normally an involuntary bodily function. Participants are instructed to synchronize their breathing rate, heart rate, and blood pressure rhythms in a way that enhances heart rate variability [23]. Maximal increases in heart rate rhythms are commonly observed at the ~6 breaths per minute breathing rate [24]. Acute increases in heart rate variability during the biofeedback training are thought to increase baseline heart rate variability and improve blood pressure reflex (baroreflex) and cognitive, emotional function, and self-regulation [25,26]. Similar baroreflex stimulation cannot be achieved during spontaneous breathing [27] or during paced breathing at faster frequencies e.g., ten [28], twelve [29], fifteen [30], or sixteen breaths per minute [28].

Previous studies show that resonance frequency breathing with heart rate variability biofeedback can reduce anxiety [31], posttraumatic stress, and depressive symptoms [16]; cardiovascular symptoms [32]; pain, insomnia and respiratory symptoms [33]; and executive functioning [34] and can lead to a better ability to regulate one’s emotions, e.g., when trying to actively reduce the intensity of a negative emotional state [35]. Preliminary findings in adult cancer patients, including breast cancer survivors, suggest that heart rate variability biofeedback can be effective for alleviating pain and fatigue and improving the quality of sleep and psychological outcomes [36]. The majority, but not all, of these studies report significant improvements in symptoms. For example, one study compared a mixed sample of cancer patients, including breast cancer patients, to healthy controls and found trends towards improvements in anxiety, stress-related symptoms, and fatigue [37]. While variable intervention duration may play a role in obtaining significant reductions in distress-related outcomes, an earlier study using a small sample of six breast cancer survivors found significant improvements in stress-management outcomes at early stages of a the 6-week heart rate variability biofeedback intervention [38].

The current study investigated the effects of a heart rate variability biofeedback intervention on some of the common cancer-related symptoms reported by breast cancer survivors using a longitudinal design with one-month and six-month follow-up testing. Our primary outcome measures were baseline heart rate variability, sleep quality, hot flush and night sweat frequency, and emotion regulation. These were based on the strength of results found in previous heart rate variability biofeedback studies and the potential of the intervention for addressing the problem of vasomotor symptoms non-invasively in the breast cancer population. Preliminary heart rate variability biofeedback studies involving cancer survivors show that sleep improvements are amongst the strongest effects [36].

To our knowledge, our study is the first study to examine the effectiveness of heart rate variability biofeedback on psychological symptoms and menopausal symptoms, and in a longitudinal manner in breast cancer survivors. Preliminary evidence suggests that slow-paced breathing (without biofeedback), including the daily practice and application of paced breathing during hot flush onset, can be effective for the management of hot flushes and night sweats [39,40]. As a secondary interest, we wanted to assess whether heart rate variability biofeedback would also improve other common psychological distress symptoms reported by breast cancer survivors, including anxiety and depression, perceived stress and posttraumatic stress, as well as potential positive impact on perceived cognitive function and fatigue.

The present study employed methods which are currently considered optimal, practical, and effective for paced breathing and heart rate variability biofeedback interventions. We used a 4-week heart rate variability biofeedback training protocol (e.g., Ref. [26] with a fixed resonance frequency of 6 breath cycles per minute (0.1 Hz) [41]. Participants used an equal inhale/exhale ratio [42,43] and performed nasal breathing during the intervention [23,44]. To help assess the effectiveness of the intervention and address the severe lack of adequate active control conditions involving breathing [24] we included an active control group who performed paced breathing at 12 breaths per minute (0.2 Hz) which lies outside of the slow breathing band, 4–10 breath cycles per minute (0.07–0.16 Hz) [45]. While small magnitude resonance effects may be possible, similar resonance effects were not expected due to a significantly shorter temporal window [24]. We also included a third group, a waitlist control group, who completed no intervention.

We predicted that following the 4-week intervention, the intervention group would show enhanced baseline heart rate variability and report an improved quality of sleep, a reduced frequency of hot flushes and night sweats, and improved emotion regulation. We also expected reduced psychological distress and improved cognitive function and fatigue symptoms. We expected the positive effects to be present at the 6 months follow-up. We predicted no pre- to post-improvement in heart rate variability, vasomotor symptoms, or sleep quality in the active control group. Some changes in psychological distress were anticipated, e.g., due to possible effects on interoception or due to meditative aspects of the practice [46,47]. We predicted no changes in any of the assessed measures in the waitlist control group across assessments.

## 2. Materials and Methods

### 2.1. Participants

**Figure 1:**
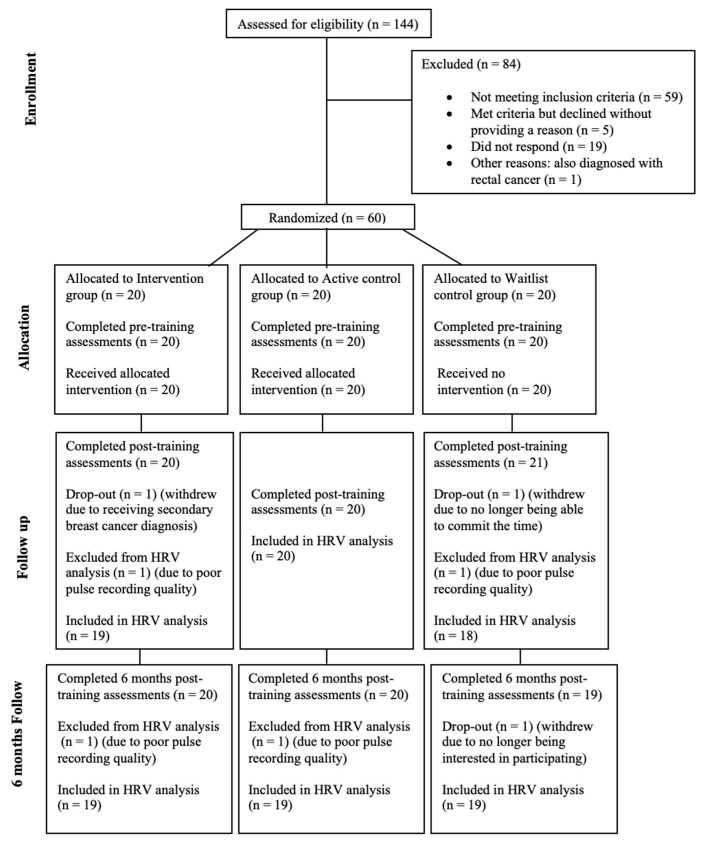
CONSORT diagram for heart rate variability biofeedback in breast cancer randomized controlled trial.

A total of sixty women were included in the study. (One participant had a secondary diagnosis of breast cancer. We included this participant in this trial who had already been allocated, as excluding them did not change the pattern of results.) The mean age was 49, and the age range was 36–61. Figure 1 depicts the CONSORT diagram. Participants were recruited between the 22 July 2022 and the 1 August 2023 on a voluntary basis via advertisements on social media platforms and relevant support groups, including Building Resilience in Breast Cancer Centre. Participants received a payment of GBP 100 in Amazon vouchers for completing all phases of the study.

### 2.2. Inclusion Criteria

The inclusion criteria were: having a primary diagnosis of breast cancer with chemotherapy as part of treatment, being between 18 and 65 years of age and between 6-months to 60-months post active treatment upon recruitment, and women could be taking regular hormone replacement or blocking therapy medications (e.g., Tamoxifen) or receiving target treatment(s) (e.g., Herceptin injections). The inclusion criteria also required participants to be experiencing sleep difficulties, hot flushes/night sweats, and cognitive difficulties to meet study criteria, such that they significantly affected their quality of life at the time of study recruitment. Participants were excluded if they were suffering any serious neurological or cardiovascular diagnosis that may affect the heart rate variability baseline, if they were on medication known to modulate heart rate variability (e.g., calcium channel blockers or β-blockers), or if they had an active seizure disorder, implantable cardioverter defibrillator in situ, substance misuse, or severe psychiatric diagnosis. Recruits could not be currently practicing breathing exercises/techniques, e.g., yogic breathing, Qi Gong Breathing, etc.

### 2.3. Power Analysis

An a priori power analysis was performed using G*Power Version 3.1 [48] to determine the minimum sample size required to test our hypotheses for repeated measures design with within-between interaction to achieve statistical power of 0.8 with an alpha of 0.05 and small effect size (*d* = 0.2). The results indicated that the minimum sample size required is 54 participants. The obtained sample of 60 participants was therefore adequate for testing our hypotheses.

### 2.4. Design

The study followed a randomized wait-list controlled trial design with three arms: an intervention arm, an active control arm, and a waitlist control condition. Self-report and physiological (i.e., heart rate variability) measures were collected at three phases: baseline (Time 1), post-intervention (Time 2), and 6 months post-intervention (Time 3). The intervention period lasted for 4 weeks. Outcome measures were collected within a one-week period for every specified phase, at −1 (pre-intervention week), +5 (post-intervention week), and +25 (six months post-intervention week). (See Figure 1.) Ethical approval was granted by the Department of Psychological Science Research Ethics Committee at Birkbeck College (ref. 2122088), and the study was pre-registered with the ISRCTN registry (trial ref. ISRCTN95964384).

### 2.5. Materials

A series of self-report questionnaires were self-administered by participants online via Gorilla Experiment Builder [49]. All the scales administered are widely used in breast cancer research showing good validity and reliability. Additionally, the following self-report measures were used to collect sociodemographic and lifestyle information found in Table S1, and clinical and information, including the breast cancer diagnosis and treatment details found in Table S2: A 28-item General Demographic Questionnaire (GDQ) (adapted from Ref. [50]).

#### 2.5.1. Primary Outcome Measures

Heart Rate Variability Indices: Time-domain heart rate variability measures of interest included the root mean square of successive RR interval differences (RMSSD), and frequency-domain measures included high frequency power (HF-power; 0.15 to 0.40 Hz range) and low frequency power (LF-power; 0.04–0.15 Hz range) [51,52]. A measure of the average heart rate was obtained and reported alongside heart rate variability.

Questionnaires:

(a) Quality of sleep was assessed using a 19-item Pittsburgh Sleep Quality Index (PSQI; [53] with seven subcomponents: Subjective Sleep Quality, Sleep Latency, Sleep Duration, Sleep Efficiency, Sleep Disturbance, Sleep Medication, and Daytime Dysfunction); a global sleep quality score was calculated and used for analysis (score range 0–2,1 where lower scores indicate better sleep quality);
(b) Emotion regulation was assessed using a 10-item Emotion Regulation Questionnaire (ERQ; [54] to assess habitual tendency to use more helpful emotion regulation strategies (Cognitive Reappraisal) and less helpful strategies (Expressive Suppression)). There was a subscale range of 1–7, and higher scores mean greater use of a given strategy;
(c) The frequency of hot flushes and night sweats was assessed using the Hot Flush Rating Scale (HFRS; [55] which measures the frequency of hot flushes and night sweats for the past week); a 3-item problem rating (1—not at all to 10—very much) was included as part of the questionnaire.

#### 2.5.2. Secondary Outcome Measures

Questionnaires:

(a) Perceived cognitive function was assessed using a 37-item Functional Assessment of Cancer Therapy-Cognitive Scale (FACT-Cog, version 3, [56] which included four subscales: Perceived Cognitive Impairment (PCI) with a range 0–72, Comments from Others (CFO) with a range 0–16, Perceived Cognitive Abilities (PCA) with a range 0–28, and Impact on Quality of Life (IQL) with a range 0–16, and the global score range was calculated and used for analysis). The score range was 0–148, where higher scores indicated better cognitive function;
(b) Anxiety and depression symptoms were assessed using a 14-item Hospital Anxiety and Depression Scale (HADS; [57] for (trait) anxiety and depression symptom severity, with a range 0–21, higher scores indicate higher anxiety and depression symptoms);
(c) Perceived stress level was assessed using a 10-item Perceived Stress Scale (PSS) [58], range 0–40, where lower scores reflect lower perceived stress level;
(d) Posttraumatic stress symptoms were assessed using a 22-item Revised Impact of Events Scale for cancer care (IES-R; [59] including four subscales: Avoidance, Intrusion, and Hyperarousal subscales, with a subscale range 0–4). The total score was calculated and used for analysis (range 0–12, where lower scores indicate fewer posttraumatic stress symptoms);
(e) Fatigue was assessed using a 13-item Functional Assessment of Chronic Illness Therapy—Fatigue (FACIT-F, version 4; [60], with a range 0–52 with higher scores indicating less fatigue).

### 2.6. Heart Rate Variability Baseline Recording Equipment

Pulse data recording was obtained using the following technology: a portable photoplethysmography Bluetooth ear sensor with a sampling rate of 120 Hz (HeartMath^®^, Boulder Creek, CA, USA) which worked in combination with a smartphone app: the Inner Balance App (Version 3.18.0.1241); [61]. Pulse rate inter-beat intervals (in milliseconds) were extracted using EmWave Pro software (Version 3.10.0.11205); [62] and then processed using the gold standard heart rate processing software Kubios (Version 2.1); [63].

The heart rate variability baseline recording took place via the video-conferencing platform Zoom [64] between 10am and 5pm for all participants at all follow-up points. The recording was always conducted by the same female experimenter in the same office room, with the same background, using the background blur feature in Zoom. The experimenter always maintained a neutral appearance (no make-up, glasses, or other accessories, and neutral clothing colors) and participants could only view the experimenter’s torso in the frame.

Participants were asked to refrain from (1) caffeinated drinks on the day prior to assessment, (2) vigorous exercise or heavy meals for two hours prior to session, (3) five or more alcoholic drinks in the 24 h before the session, and (4) nicotine for six hours before the session [65,66].

### 2.7. Heart Rate Variability Training Protocol

An adapted 4-week fixed frequency heart rate variability biofeedback training protocol [67] was used.

*Intervention:* Participants used the same specified pre-set breathing pace for the duration of the 4-week intervention, i.e., resonance frequency breathing at 6 breaths per minute. The 4-week training included 5 training days per week: in week 1—10 min twice daily, in week 2—15 min twice daily, in week 3—20 min twice daily, and in week 4—20 min twice daily. The two days without training could be consecutive or non-consecutive. Heart rate variability biofeedback training was self-administered by participants who received close (remote) active monitoring and support from the trial coordinator throughout the intervention phase. The coordinator emailed participants weekly to check in to ensure training was performed safely, with good technique, and to inquire and resolve any relevant challenges, e.g., to help plan training sessions.

*Active control*: Participants in the active control group completed 4 weeks of heart rate variability biofeedback training but trained at 12 breaths per minute. They received identical general briefing, training instructions, and tools, except for the breathing pace they used during the 4-week training period.

*Waitlist control:* Waitlist participants completed no intervention but completed self-report and baseline heart rate variability assessments at the same time intervals as the intervention and active control groups. Between assessments, waitlist participants were instructed to engage in their normal routine. After completing their 6 months follow-up, for ethical reasons, including compensation for the delay in the opportunity to receive the intervention, participants received all the necessary training instructions and materials (i.e., the 4-week training protocol and pulse sensor) enabling them to complete the 4-week heart rate variability biofeedback intervention independently. These waitlist participants kept the pulse sensors as a gift.

### 2.8. Procedure

Study recruits confirmed eligibility via email and telephone communication with the primary study researcher. Eligible recruits confirmed their interest and provided signed consent electronically, after which they were assigned a participant ID number. Next, each participant was randomly allocated to one of the following three groups: intervention group (*n* = 20), active control group (*n* = 20), or waitlist control group (*n* = 20).

Sealed envelope software version 1.22.0 [68] was used to randomly allocate participants to the three groups with a 1:1:1 ratio, using block sizes of 3, 6, and 9 in the design. To prevent any biases and ensure random allocation during recruitment, participants were recruited on a rolling basis and group allocations were concealed for the researchers until the enrolment of the total sample was complete. Participants in the intervention and active control groups were blind to the condition, told they were allocated to the intervention group, and received identical treatment and instructions. Breathing pace during intervention was the only difference, i.e., 0.1 Hz or 0.2 Hz. Participants were posted the Bluetooth sensor device along with pre-paid return materials to their home address. Next, they were sent an online link to complete the set of questionnaire measures, including anxiety and depression, cognitive function, sleep quality, and vasomotor symptoms, etc. Informed consent for the questionnaire battery completion was also obtained online at every follow-up point. Later in the same week, participants completed two 1 h Zoom-based sessions with the trial coordinator on two separate days (session 1—heart rate variability baseline recording, session 2—paced breathing and heart rate variability biofeedback training).

Heart rate variability baseline recording took place remotely via the online platform Zoom. Participants ensured they had a private space for the online session, typically using a private room in their home. A 5 min resting baseline was recorded, and a 4 min segment (00:00:30–00:04:30) was used for analysis [51]. The segment was reduced in this fashion to avoid potential reactivity and movement artifacts associated with recording initiation and completion. The baseline session lasted approximately 1 h and included Inner Balance App login and pulse sensor set-up. During the recording, participants were instructed to sit in a comfortable upright posture with arms and legs uncrossed and shoulders down and relaxed. Their task was to rest and clear their mind for 5 min, to minimize any movement, and to breathe normally (spontaneously).

To ensure privacy for the participant during the baseline recording, the experimenter turned off their computer microphone and exited the camera view, leaving only the blurred office background on the computer visible on the participant’s end.

The HeartMath Bluetooth pulse sensor placed on left ear lobe coupled with the Inner Balance smartphone App enabled participants to perform the heart rate variability biofeedback training. During training sessions, participants followed a visual breath pacer on their smartphone screen, i.e., a thick graded line moving up and down. The ear sensor captured inter-beat intervals, transforming them into frequency measures of the heart rate variability spectrum (very low frequency/low frequency/high frequency). The biofeedback element was represented by heart rhythm coherence where coherence was defined by a high-amplitude peak (typically occurring at ~0.1 Hz) in the low frequency (LF) band of the heart rate variability power spectrum. An algorithm quantified coherence by identifying the maximum peak in the 0.04–0.26 Hz range, calculating the integral in a 0.030 Hz window to determine peak power, then calculating the total power of the whole heart rate variability spectrum. The coherence ratio is calculated using the following formula: (Peak Power/(Total Power—Peak Power) [69]. For the participant, the coherence level was represented and differentiated by the color of the graded line which changed dynamically during practice. The color feedback included the following levels: green—high coherence, blue—medium coherence, and red—low coherence.

For the independent 4-week training, participants were advised to plan and fit the two daily training session into their routine, i.e., no set training times. They were advised to adopt a comfortable upright posture with arms and legs uncrossed and feet placed flat on the floor to ensure comfort and good technique. They were also advised on and shown how to relax their body (progressive muscle relaxation and breathing regulation strategies) and on how to manage any discomfort, including hyperventilation, in case they felt tense, restless, or anxious before or during training sessions. A leaflet summarizing this advice was provided and they were invited to contact the trial coordinator in case of any relevant issues. The trial coordinator monitored training recordings using EmWave Pro software [62], checked in with each participant via email once a week with training settings updates or in the case that no data recordings were observed for more than two days.

### 2.9. Data Analysis

Prior to statistical analysis, heart rate variability parameters were recorded through EmWave Pro software [62] to obtain inter-beat intervals. Each pulse trace was then processed individually in Kubios (Version 2.1) [63]; and inspected for outlier intervals (motion artifacts, ectopic beats) through visual inspection [51]. Intervals exceeding the local median by 200 msec were considered noise and were excluded from analysis [63]. To help ensure normal distribution, natural log-transformed HF and LF power measures (ln HF and ln LF) were used following Task Force [51] recommendations. Raw RMSSD (msec) values were extracted and used for analysis. Lower RMSSD baseline values are more common than higher values in pathology, and we aimed to be as inclusive as possible considering that individual heart rate variability baseline can be highly variable [70]. HRV parameters were examined for outliers using boxplots and histograms, and Winsorization was applied to help normalise the distribution. Winzorization was applied to 10% of RMSSD, ln LF, and ln HF observations (i.e., in the 95th and 5th percentiles) at each of the three time points. (Six heart rate variability traces were excluded from the analysis due to poor recording quality (post-intervention: one in the intervention group, two in the waitlist control group at post-intervention, and one in each group at the 6 months follow-up). One outlier for the Fatigue score was identified at the baseline and was removed from the analysis due to significantly skewing the results.).

#### Statistical Analysis

Statistical analysis was performed using the Statistical Package for the Social Sciences (IBM SPSS, Version 29; [71]). To examine the effects of training on resting HRV and self-reported questionnaires over time, Linear Mixed Effect Models (MLMs) with a first-order autoregressive structure (AR (1)) were used. Time (baseline, post-training, 6 months post- training), group (intervention group, active control group, waitlist control group) and time × group interaction were entered as fixed effects in the MLM models. Participants were entered as random effects. BMI was added to the model as a covariate for the analysis of heart rate variability and heart rate. Model (parameter) estimation was conducted using a maximum likelihood method. Cohen’s *d* was calculated using the following formula: *d* = 2 × √(F/df). Significant time x group interactions were followed up using within-subject one-way analyses of variance (ANOVAs) with time as a repeated measures factor (3 levels: baseline, post-intervention, 6 months). Greenhouse–Geisser values were reported in the case of sphericity assumption violation. Polynomial contrasts were conducted to test for linear and quadratic trends and Post-hoc Simple Effects contrasts with Bonferroni-corrected values were used for comparisons. To further investigate changes in vasomotor symptoms, specifically frequency across time, Pearson’s Chi-square analysis with a significance level of 0.05 was performed on the number of cases who reported *cessation* of night sweats.

## 3. Results

Table 1 shows the sociodemographic characteristics of the sample. Sixty women with a history of primary breast cancer with the mean age of 49 years at recruitment (SD = 6.2, ranging from 36 to 61) took part in the study. Most participants were highly educated, married women of white background. Please see Table S1 for details of breast cancer diagnosis and treatment and other clinical history.

The participants in the intervention and active control groups completed an average of 97% of the allocated breathing training (see Table 2) demonstrating the feasibility of heart rate variability biofeedback in community settings in breast cancer survivors (Neither training group reported any significant discomfort or other adverse effects during their training. On occasion, some participants reported challenges with finding time for training, feeling frustrated, sleepy or distracted by thoughts during training sessions. These challenges were discussed and addressed with the trial coordinator, who advised participants on training session planning and training environment set-up to avoid physical discomfort or distractions.).

### 3.1. Baseline Heart Rate Variability Indices

Univariate analysis of variance confirmed that there were no baseline differences between groups in HR (F (2, 57) = 2.09, *p* = 0.133), RMSSD (F (2, 57) = 1.82, p = 0.171), ln LF power (F (2, 57) = 1.26, *p* = 0.292), ln HF power (F (2, 57) = 2.41, *p* = 0.099), or BMI (F (2, 57) = 0.000, *ns*) (See Table 3).

*Heart Rate Variability:* An improvement in the baseline heart rate variability was observed in the intervention group. For summary of heart rate variability changes across time, see Table 3.

*Ln LF power:* There was a significant time x group interaction for ln LF power (F (4, 103.89) = 2.862, *p* = 0.027, *d* = 0.33), which was qualified by a linear trend showing increases in ln LF power across time in the Intervention group only (*p* = 0.004).

*RMSSD:* The interaction of time x group fell short of significance for RMSSD (F (4, 115.30) = 2.176, *p* = 0.076, *d* = 0.27).

*Ln HF power:* The time x group interaction fell short of significance for ln HF power, (F (4, 116.65) = 2.161, *p* = 0.078, *d* = 0.29).

*Average Heart Rate:* There was no time x group interaction for average heart rate, (F (4, 114.99) = 1.919, *p* = 0.112, *d* = 0.28).

### 3.2. Overall Sleep Quality:

**Figure 2:**
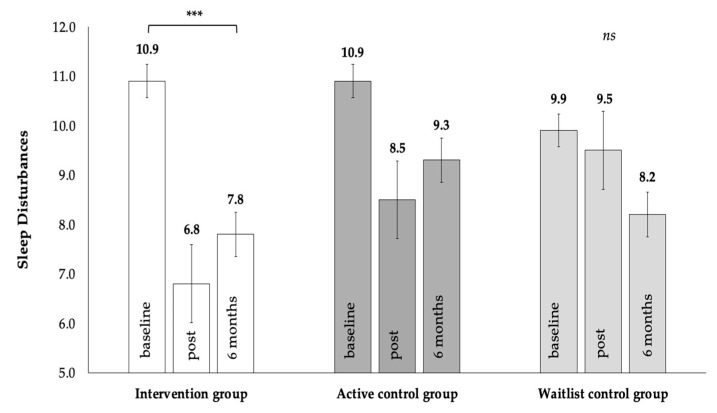
Self-reported sleep quality at baseline, post-intervention, and at the 6 months follow-up; *** *p* < 0.001, *ns p* > 0.05.

A significant group x time interaction, F (4, 88.04) = 4.87, *p* = 0.001, *d* = 0.43, which was qualified with a significant linear trend (*p* < 0.001), showed sustained improvements in sleep quality from baseline to post-intervention and from post- to six-month follow-up (*ps* < 0.001) in the intervention group (Figure 2). The active control group showed a significant quadratic trend (*p* = 0.012) reflecting improvements from the baseline to post-intervention (*p* = 0.003), which was not sustained at six months’ follow-up after Bonferroni correction. The waitlist group showed no significant changes across time (see Table S3).

### 3.3. Night Sweats and Hot Flushes

**Figure 3:**
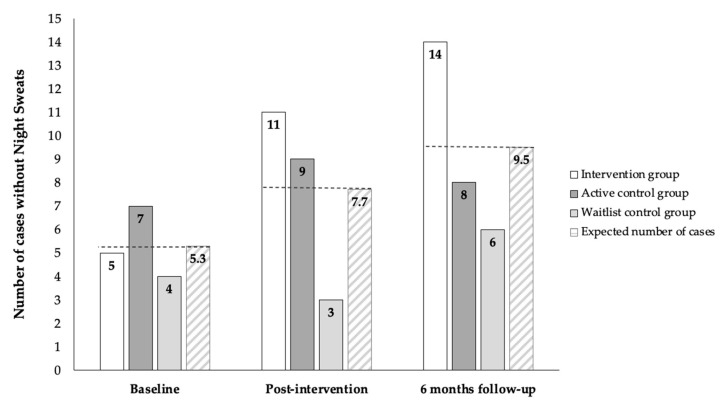
Number of cases reporting not having night sweats at baseline, post-intervention, and 6 months follow-up as compared to expected number of cases at each time-point.

***Night Sweats*:** Chi-square analysis showed that women in the intervention group were significantly more likely to report the cessation of night sweats post-intervention (*X*^2^ (2, *N* = 60) = 7.33, *p* = 0.02, Cramer’s V = 0.35), and at the 6 months’ follow-up, (*X*^2^ (2, *N* = 59) = 6.44, *p* = 0.04, Cramer’s V = 0.33). The number of cases reporting no night sweats in the intervention group went up from 5 at the baseline to 11 cases post-intervention, and up to 14 no-night-sweats cases at 6 months. In contrast, the number of cases without nights sweats in the active control group bordered around the expected frequency at each assessment point. The waitlist group was consistently below the expected frequency (see Figure 3).

*Hot Flushes:* There was no group x time interaction (F (4, 82.50) < 1, *ns*).

### 3.4. Psychological Well-Being, Cognitive Function and Fatigue

Table S2 shows the descriptive statistics for psychological, cognitive, and fatigue symptoms.

*Anxiety:* The group x time interaction, F (4, 82.51) = 2.99, *p* = 0.023, *d* = 0.44 showed an improvement in anxiety in the intervention group. A significant quadratic trend (*p* = 0.002) and pairwise comparisons showed significant improvement post-intervention (*p* = 0.003) which was not sustained at the 6 months follow-up (*p* > 0.05). The active control and waitlist control groups showed no significant trends across time. ***Depression:*** No group x time interaction was found, F (4, 75.78) = 2.02, *p* = 0.101, *d* = 0.33).

*Perceived Stress:* A significant group x time interaction, (F (4, 95.94) = 3.65, *p* = 0.008, *d* = 0.41) showed changes in perceived stress in the intervention and active control groups across time (*p*s < 0.03). The waitlist control group reported no changes, *ps* > 0.05. *Posttraumatic Stress:* A significant group x time interaction, F (4, 108.54) = 4.359, *p* = 0.003, *d* = 0.38, found changes in posttraumatic stress symptoms in the intervention and active control groups across time with significant reductions from the baseline to 6 months follow-up (*ps* < 0.005). No changes were observed in the waitlist control group, *ps* > 0.05. *Cognitive Reappraisal:* There was no group x time interaction, F (4, 114.12) = 1.69, *d* = 0.24, *p* = 0.158. *Cognitive Function*: There was a significant group x time interaction, F (4, 80.76) = 3.52, *p* = 0.003, *d* = 0.45 which was qualified by a significant linear trend for the intervention and active control groups showing reductions across time (*ps* < 0.005). No changes in perceived cognitive function were evident in the waitlist control group (*ps* > 0.05). ***Fatigue:*** There was a significant time x group interaction, F (4, 83.85) = 2.56, *p* = 0.044, *d* = 0.35. Significant linear trends indicated improvement in fatigue symptoms across time in the intervention group (*p* = 0.004) and active control group (*p* < 0.001). No significant changes were reported in the waitlist groups (*ps* > 0.05). (Table S3).

## 4. Discussion

To our knowledge, our study is the first to assess the effectiveness of a 4-week heart rate variability biofeedback intervention on baseline heart rate variability and a range of common cancer-related symptoms affecting female breast cancer survivors. We compared observations from breast cancer survivors who completed an intervention known to improve psychological and physical well-being (i.e., resonance frequency breathing with heart rate variability biofeedback) to active control intervention and waitlist control conditions. As predicted, participants in the intervention group showed significant improvements in heart rate variability and reported better sleep quality and vasomotor symptoms, including the cessation of night sweats, as well as improvements in anxiety, perceived stress, post-traumatic stress, cognitive function, and fatigue after the 4-week intervention. Importantly, a number of these improvements remained at the 6 months follow-up. The active control group who also performed daily paced breathing exercises for 4 weeks showed similar sustained improvement in perceived cognitive function, stress-related symptoms, and fatigue and reported smaller and less sustained improvements in quality of sleep.

Consistent with previous findings [36] our results showed a significant sustained enhancement in baseline heart rate variability (ln LF power) only in the intervention group. An increase in ln LF power, specifically, is considered as evidence of improvement in baroreflex activity/sensitivity [29,72]. It is noteworthy that the intervention group also showed trends towards sustained increase in the other heart rate variability parameters (i.e., RMSSD and ln HF power) which are also commonly interpreted as improvement in vagally mediated heart rate variability [51]. Considering the magnitude of autonomic dysregulation and multimorbidity in breast cancer, longer interventions may help to promote further improvements in heart rate variability in this population. However, systematic reviews suggest that training time (e.g., number of sessions) does not have a significant impact on the effect sizes of heart rate variability changes, behavioral and self-report outcomes [16,26]. In keeping with previous findings which assessed the effects of 12-breaths-per-minute (0.2. Hz) breathing [29,73] women in the active control group showed no evidence of heart rate variability enhancement. This makes 0.2 Hz a useful active control for not only short- or single-session assessments, but also for longer-term heart rate variability biofeedback interventions. Women in the active control group reported some improvements in perceived stress, fatigue, and cognitive function, with some relatively smaller improvements in post-traumatic stress symptoms and sleep quality. In contrast, women who received no intervention (wait list control group) reported no significant changes in any of the assessed symptoms over time. Taken together, our findings suggest that heart rate variability enhancement stimulated via resonance frequency breathing with heart rate variability biofeedback may indeed play an important role in strengthening homeostatic mechanisms, leading to improved autonomic regulation and stress resilience in breast cancer survivors.

The intervention group reported significant improvements in sleep quality after the 4-week intervention. Amongst the changes across various aspects of sleep quality, they reported a 50% reduction in the time taken to fall asleep after the intervention (from ~45 min at the baseline, down to ~23 min post-intervention, relapsing at the 6 months follow-up to ~40 min; see Table S3). These observations are consistent with previous findings which show that sleep initiation problems are linked to sympathetic overactivation and decreased arousal threshold [74,75]. Previous studies show that 15–20 min of resonance frequency breathing before bedtime is associated with shorter slow-wave sleep onset, better sleep efficiency, and subjective sleep quality as compared to control conditions, including 12-breaths-per-minute breathing and spontaneous breathing [76,77,78]. Our participants completed two daily training sessions and generally one in the morning and one in the afternoon. This suggests that heart rate variability biofeedback practice has lingering and cumulative effects on the autonomic nervous system. Breathing and the neurophysiology of sleep are also linked where improvement in hypothalamic function and cardiorespiratory synchronization is proposed to mediate improvements in sleep [79]. The hypothalamus has a central role in homeostasis maintenance and, amongst various functions (posterior), the hypothalamus helps regulate breathing and oxygen and carbon dioxide levels in the body [80] when we are awake, asleep, or under stress [81]. Heart rate variability biofeedback likely produced significant bottom-up influences on regulatory functions of the hypothalamus.

Women in the intervention group were significantly more likely to report cessation of night sweats following the 4-week intervention. Improvements in baroreflex function, i.e., vascular and circulatory function, could be the mechanism by which heart rate variability biofeedback affects thermoregulation [82]. Vasomotor symptoms are associated with decreased communication between the vagus nerve and nucleus tractus solitarius, which plays an important role in relaying visceral signals, including respiratory and cardiovascular information [83,84]. Our findings suggest that improving baroreflex sensitivity via paced breathing with heart rate variability biofeedback intervention appears to improve thermoregulatory disturbance associated with menopausal vasomotor symptoms in breast cancer.

We found no significant changes in self-reported emotion regulation. However, we found an improvement in self-reported anxiety, perceived stress, and post-traumatic stress in the intervention group, which is evidence of improved psychological well-being and distress management capacity. Heart rate variability biofeedback training and improvement in baroreflex function (i.e., ln LF power) likely had an impact on the arousal mechanisms involved in emotion and somatosensory regulation, i.e., amygdala–hypothalamic–pituitary–adrenal axis communication [85,86]. These observations are in line with the neurovisceral integration model [87] which assumes a bidirectional connection between the heart and the brain and links cognitive, emotional, and cardiac regulation to the central autonomic network (CAN) which is thought to underly self-regulation [88,89]. The model suggests that the amygdala is under tonic inhibition of the medial pre-frontal cortex, which allows better emotional and cognitive regulation [15,90]. Neuroimaging evidence links increased structural and functional medial prefrontal cortex and amygdala connectivity and improved emotional regulation [90,91] in support of the neurovisceral integration hypothesis. While effective self-regulation involves autonomic changes and top-down cognitive reappraisals, the behavioral ability to influence one’s bodily state including voluntarily breath control plays an important role [26,92].

Some reductions in stress-related symptoms were also reported in the active control group. We believe that this was most likely due to increased awareness, interoception, and behavioral effects of paced breathing. Studies using faster paced breathing techniques are very scarce; however, a recent systematic review of paced breathing randomized controlled trials indeed found that all techniques which involve voluntary regulated breathing including faster paced ones are associated with reductions in self-reported stress, especially in high-anxiety populations [93]. Bentley et al. [93] hypothesized that breathing at any pace that is different from one’s normal breathing (i.e., paced vs. spontaneous) may provide stress relief, and that a sense of control from the acquired ability to control one’s breathing and internal bodily states could be one potential mechanism. A recent meta-analysis [94] compared paced breathing to non-breathing control interventions e.g., mindfulness-based techniques for stress reduction and found similar reductions in self-reported stress across interventions implicating attention-based influences in stress reduction in paced breathing interventions. Mindfulness-based stress reduction is associated with an improvement in the symptoms of insomnia [95] which may help explain some improvement in the quality of sleep in the active control group.

The intervention and active control groups reported comparable improvement in perceived cognitive function and fatigue which was sustained at 6 months. While fatigue and cognitive impairment are closely related in breast cancer [96], mechanisms causing and maintaining cognitive dysfunction in breast cancer are poorly understood with various other potential factors being linked, including insomnia, anxiety, depression, cardiovascular disease, menopausal symptoms, and adjuvant treatments [97,98]. Breathing exercises, especially slow-paced breathing, have previously been shown to improve cognitive function in cardiovascular disease with effects also linked to baroreflex activity [99]. The active control group in our study showed no heart rate variability enhancement. Like the intervention group, active control participants were trained to breathe nasally during their training sessions, and nasal breathing could have resulted in improved self-reported cognitive function. Neuroimaging research indeed shows that nasal breathing is linked to better cognitive performance and enhanced functional connectivity in brain regions associated with working memory functions [100,101]. Other, particularly behavioral and interoceptive, factors could have also played a positive role [102]. This also suggests that despite other intervention-specific effects (baseline heart rate variability enhancement, improvement of vasomotor symptoms, sleep quality, and anxiety) similar mechanisms, i.e., interoceptive and behavioral aspects of the resonance frequency intervention may be key for improvements in stress-related symptoms and energy levels in resonance frequency breathing intervention.

### Limitations and Future Research

Our sample consisted mainly of highly educated white women which could bias our outcomes. Future studies must encourage recruits from a wider cultural, ethnic, and social community to take part to help better assess the feasibility of heart rate variability across community settings. We excluded women with any cardiovascular diagnoses due to potential adverse effects and challenges with assessing the impact of the intervention on heart rate variability. However, the impact of the intervention on cardiovascular function in women with cardiovascular diagnoses is important to explore considering the increased risk and prevalence of cardiovascular conditions in women with breast cancer diagnosis history.

To help develop heart rate variability biofeedback interventions for breast cancer populations, future studies should consider collecting additional physiological (breathing rate, blood pressure, sleep-wake actigraphy) and endocrine measures (cortisol markers). This would help understand the effects of the intervention on the autonomic function and help uncover potential mechanisms. Additional tools, such as diaries and phone applications, may also be useful for monitoring sleep changes and hot flushes to help develop recommendations for breast cancer survivors, e.g., optimal training time, duration, frequency, and specific symptom management. Additionally, to help determinine the utility of heart rate variability biofeedback for distress management in breast cancer, replicating this study on younger and older breast cancer survivors may be useful as women of different age groups may show different levels of vulnerability to mood and anxiety-related problems. For example, evidence shows that younger women are more likely to experience severe fear of cancer recurrence [4] while older breast cancer survivors show higher depression rates [103].

Further research is necessary as there is presently no guidelines for the *optimal* or *tailored* application of heart rate variability biofeedback for psychological or physical symptoms across populations. It appears to be a relatively safe and simple intervention for self-administration. Professional support and guidance are desirable. For example, integrating heart rate variability biofeedback into existing evidence-based psychological approaches may be most optimal due to important links between cognition, behavior, and physiology in distress management [104,105].

## 5. Conclusions

Our findings show that heart rate variability biofeedback can enhance baseline heart rate variability and improve a range of cancer-related symptoms in breast cancer survivors with sustained effects. It is important to note that baroreflex plays a key role in autonomic cardiovascular control [106], hence its enhancement via heart rate variability biofeedback could serve to mitigate and prevent some cardiovascular disease risks and promote better cardiovascular health in breast cancer survivors.

## Figures and Tables

**Table 1:** Means and standard deviations for self-reported demographic and lifestyle characteristics at baseline.

	Intervention Group (*n* = 20)		Active Control Group (*n* = 20)		Waitlist Control Group (*n* = 20)	
**Sociodemographic**	Mean age = 49.6 (6.2) (Min = 39, Max = 61)		Mean age = 49.6 (6.2) (Min = 36, Max = 59)		Mean age = 49.2 (6.2) (Min = 36, Max = 59)	
Age (years)						
**Education**	**No. of cases**	**%**	**No. of cases**	**%**	**No. of cases**	**%**
Secondary education	2	10	0	0	1	5
Further education	2	10	5	25	4	20
Higher education	16	80	15	75	15	75
**Work**						
Employed	13	65	16	80	12	60
Self-employed	1	5	0	0	1	5
Volunteering work	0	0	1	5	1	5
Not in work	5	25	2	10	4	20
Other (on sick leave, retired etc)	1	5	1	5	2	10
**Ethnicity**						
White	19	95	20	100	20	100
Asian	1	5	0	0	0	0
**Marital status**						
Married	15	75	14	70	14	70
Cohabiting with a Partner	0	0	1	5	2	10
Divorced	1	5	3	15	1	5
Separated	0	0	1	5	0	0
Single	3	15	1	5	3	15
Widowed	1	5	0	0	0	0
**Health and lifestyle information**						
Height (in cm)	168.0 (7.1)		168.1 (7.1)		168.5 (7.0)	
Weight (in kg)	75.8 (17.1)		76.3 (16.9)		77.7 (5.8)	
Body Mass Index (BMI)	26.8 (5.8)		27.0 (5.8)		27.4 (5.8)	
**Smoking status**						
Current smoker	0	0	0	0	1	5
Former smoker	8	40	5	25	10	50
Electronic cigarette user	0	0	0	0	1	5
Never smoked	12	60	15	75	8	40
**Menopausal status at diagnosis ^1^**						
Premenopausal	13	65	9	45	9	45
Perimenopausal	4	20	2	10	3	15
Menopausal	0	0	1	5	0	0
Postmenopausal	2	10	7	35	4	20

^1^ Six women did not provide their menopausal status (one in the intervention group, one in the active control group, and four in the waitlist control group).

**Table 2:** Heart rate variability biofeedback practice time during the 4-week intervention period.

Group.	Average Time (in Minutes)	SD	(%)	Min	(%)	Max	(%)
Intervention group	**641**	(31)	**98**	548	84	690	106
Active control group	**627**	(60)	**96**	470	72	781	120

**Table 3:** Changes in baseline heart rate and heart rate variability at baseline, post-intervention, and 6 months’ follow-up.

	Intervention Group			Active Control Group			Waitlist Control Group		
	Baseline	Post	6 Months	Baseline	Post	6 Months	Baseline	Post	6 Months
	(*n* = 20)	(*n* = 19)	(*n* = 19)	(*n* = 20)	(*n* = 20)	(*n* = 19)	(*n* = 20)	(*n* = 18)	(*n* = 19)
	Mean (SD)			Mean (SD)			Mean (SD)		
**HR**	**77.92** (9.72)	**78.81** (10.03)	**74.19** (12.10)	**73.93** (14.68)	**77.77** (12.55)	**77.63** (13.52)	**68.25** (12.42)	**68.65** (10.45)	**67.40** (13.00)
**RMSSD**	**35.66** (28.14)	**54.41** (34.90)	**52.72** (33.95)	**50.71** (39.37)	**55.47** (37.33)	**44.43** (25.69)	**53.57** (26.54)	**43.91** (20.90)	**55.76** (29.97)
**Ln HF power**	**5.39** (1.24)	**6.13** (1.56)	**5.99** (1.28)	**6.01** (1.50)	**6.09** (1.43)	**5.67** (1.30)	**6.29** (1.04)	**6.11** (1.09)	**6.47** (0.94)
**Ln LF power**	**5.78** (1.09)	**6.57** (1.18)	**6.76** (1.31)	**6.20** (1.14)	**6.10** (1.33)	**6.17** (1.32)	**6.39** (1.12)	**6.23** (1.12)	**6.55** (1.37)

## Data Availability

Data are contained within the article and Supplementary Materials.

## Supplementary Materials

The following supporting information can be downloaded at: https://www.mdpi.com/article/10.3390/curroncol32030150/s1, Table S1: Means and standard deviations for self-reported breast cancer diagnosis and treatment history and other clinical history at baseline; Table S2: Means and standard deviations for self-report measures of psychological well-being, cognitive function, baseline, post-intervention, and 6 months follow-up; Table S3: Means and standard deviations for self-report measures of quality of sleep, fatigue, and vasomotor symptoms at pre-, post-intervention, and 6 months follow-up.
