# Supplementary material for: Heart Rate Variability Biofeedback Training Can Improve Menopausal Symptoms and Psychological Well-Being in Women with a Diagnosis of Primary Breast Cancer: A Longitudinal Randomized Controlled Trial
Source: Curr Oncol. 2025 Mar 4;32(3):150. doi: 10.3390/curroncol32030150 (PMC11941165; doi:10.3390/curroncol32030150)
Supplement: Supplementary file 1 [file curroncol-32-00150-s001.zip › Table S1 CO .docx]

**Table S1**

|  |  | | |  | | | |  |  |
| --- | --- | --- | --- | --- | --- | --- | --- | --- | --- |
| **Clinical** | **Intervention**  **group (n = 20)** | |  | **Active control**  **group (n = 20)** | | |  | **Waitlist control**  **group (n = 20)** |  |
|  |  | |  |  | |  | |  |  |
| Age at diagnosis (breast cancer) | Mean age = 46.7 (6.0) (Min = 33, Max = 58) | | | | Mean age = 45.8 (6.0) (Min = 33, Max = 58) | | | Mean age = 45.4 (6.0)  (Min = 34, Max = 54) | |
| Time since diagnosis (in months) | 37.4 (20.4)  (Min = 13, Max = 83) | | | | 37.4 (20.4)  (Min = 14, Max = 116) | | | 37.1 (20.0)  (Min = 12, Max = 67) | |
|  | **No.** | **%** | | **No.** | | **%** | | **No.** | **%** |
| **Primary breast cancer diagnosis** | 20 | 100 | | 20 | | 100 | | 19 | 95 |
|  |  |  | |  | |  | |  |  |
| **Type of breast cancer** |  |  | |  | |  | |  |  |
| Ductal Carcinoma in Situ (DCIS) | 2 | 10 | | 0 | | 0 | | 0 | 0 |
| Invasive Ductal Carcinoma (IDC) | 15 | 75 | | 10 | | 50 | | 10 | 50 |
| Invasive Lobular Breast Cancer | 0 | 0 | | 2 | | 10 | | 3 | 15 |
| Mixed IDC and DCIS | 3 | 15 | | 4 | | 20 | | 4 | 20 |
| Inflammatory Breast Cancer | 0 | 0 | | 2 | | 10 | | 2 | 10 |
| Other^a^ | 0 | 0 | | 2 | | 10 | | 1 | 5 |
|  |  |  | |  | |  | |  |  |
| **Grade of breast cancer** |  |  | |  | |  | |  |  |
| Grade 1 | 0^b^ | 0 | | 1 | | 5 | | 1 | 5 |
| Grade 2 | 8 | 40 | | 7 | | 35 | | 7 | 35 |
| Grade 3 | 11 | 55 | | 12 | | 60 | | 12 | 60 |
|  |  |  | |  | |  | |  |  |
| **Breast cancer treatment history** |  |  | |  | |  | |  |  |
|  |  |  | |  | |  | |  |  |
| **Type of treatment received** |  |  | |  | |  | |  |  |
| Chemotherapy | 20 | 100 | | 19 | | 95 | | 18 | 90 |
| Radiotherapy | 17 | 85 | | 16 | | 80 | | 16 | 80 |
|  |  |  | | |  |  | |  |  |
| **Surgical procedure** |  |  | | |  |  | |  |  |
| Mastectomy | 13 | 65 | | | 14 | 70 | | 12^c^ | 60 |
| Lumpectomy | 6 | 30 | | | 4 | 20 | | 7 | 35 |
| Mastectomy and Lumpectomy | 1 | 5 | | | 2 | 10 | | 0 | 0 |
|  |  |  | | |  |  | |  |  |
| **Time since active treatment**  **(in months)** | Mean = 26.0 (17.6)  (Min = 6, Max = 83) | | | | Mean = 25.7 (17.6)  (Min = 6, Max = 60) | | | Mean = 23.2 (14.6)  (Min = 6, Max = 56) | |
|  |  |  | | |  |  | |  |  |
| **Adjuvant therapy** |  |  | | |  |  | |  |  |
| Endocrine therapy | 19 | 95 | | | 18 | 90 | | 18 | 90 |
| Targeted therapy | 6 | 30 | | | 5 | 25 | | 5 | 25 |
| Endocrine and Targeted therapies | 6 | 30 | | | 2 | 10 | | 3 | 15 |
| None currently | 1 | 5 | | | 2 | 10 | | 2 | 10 |

Means and standard deviations for self-reported breast cancer diagnosis and treatment history and other clinical history at baseline

| **Mental health** |  |  |  |  |  |  |
| --- | --- | --- | --- | --- | --- | --- |
| Anxiety | 6 | 30 | 2 | 10 | 3 | 15 |
| Depression | 3 | 15 | 4 | 20 | 2 | 10 |
| Anxiety and Depression | 3 | 15 | 2 | 10 | 3 | 15 |
|  |  |  |  |  |  |  |
| On prescribed medication for anxiety or depression | 5 | 25 | 4 | 20 | 4 | 20 |

| **Menopausal status at diagnosis ^d^** |  |  |  |  |  |  |
| --- | --- | --- | --- | --- | --- | --- |
| Premenopausal | 13 | 65 | 9 | 45 | 9 | 45 |
| Perimenopausal | 4 | 20 | 2 | 10 | 3 | 15 |
| Menopausal | 0 | 0 | 1 | 5 | 0 | 0 |
| Postmenopausal | 2 | 10 | 7 | 35 | 4 | 20 |

| On prescribed non-hormone medication for menopausal symptoms ^e^ | 2 | 10 | 1 | 5 | 3 | 15 |
| --- | --- | --- | --- | --- | --- | --- |
| Pain medication | 0 | 0 | 1^f^ | 5 | 0 | 0 |
|  |  |  |  |  |  |  |
| History of Covid-19 diagnosis | 13 | 65 | 11 | 55 | 14 | 70 |
|  |  |  |  |  |  |  |

| **Physical exercise (min per week)** | |  |  | | |  | |  |  |  | |
| --- | --- | --- | --- | --- | --- | --- | --- | --- | --- | --- | --- |
| Aerobic exercise | | 170.9 (157.8) | | |  | | 175.2 (159.6) |  | 172.1 (160.7) | |  |
| Flexibility exercise | | 46.7 (69.7) | | |  | | 48.2 (70.0) |  | 41.8 (67.2) | |  |
| Resistance exercise | | 30.3 (54.9) | | |  | | 30.8 (55.2) |  | 28.8 (56.7) | |  |
| Breathing techniques | | 0 | |  | | | 0 |  | 0 | |  |
|  | |  | |  | | |  |  |  | |  |
|  |  |  |  |  |  |  |  |  |  |  |  |

*Note*. ^a^One participant reported IDC, DCIS and Invasive Lobular breast cancer and one IDC and Invasive Lobular breast cancer in the active control group; one waitlist participant reported Occult Breast cancer diagnois; ^b^One participant in the intervention group did not provide information on cancer Grade; ^c^One participant in the waitlist control group reported having no surgery as part of their breast cancer treatment; ^d^Six women did not provide their menopausal status (one in the intervention group, one in the active control group and four in the waitlist control group); ^e^ Two participants reported being on a low dose of antihypertensive drug such as Clonidine for managing menopausal hot flushes and sweats; ^f^One waitlist control participant reported taking Tramadol for bone stress fractures as a temporary measure at the initial assessment.
