# Supplementary material for: Heart Rate Variability Biofeedback Training Can Improve Menopausal Symptoms and Psychological Well-Being in Women with a Diagnosis of Primary Breast Cancer: A Longitudinal Randomized Controlled Trial
Source: Curr Oncol. 2025 Mar 4;32(3):150. doi: 10.3390/curroncol32030150 (PMC11941165; doi:10.3390/curroncol32030150)
Supplement: Supplementary file 1 [file curroncol-32-00150-s001.zip › Table S2 CO.docx]

**Table S2**

****Means and standard deviations for self-report measures of psychological wellbeing, cognitive function, baseline, post-intervention and 6 months follow-up
